# A circulating vaccine-derived poliovirus type 2 outbreak in a chronic conflict setting: a descriptive epidemiological study in South Sudan – 2020 to 2021

**DOI:** 10.1186/s12879-023-08758-z

**Published:** 2023-11-21

**Authors:** Ayesheshem Ademe Tegegne, Atem Nathan Anyuon, George Awzenio Legge, Melisachew Adane Ferede, Zingbondo Isaac, Kirbak Anthony Laku, Sibhatu Biadgilign, Ochan Taban David Kilo, Fabian Ndenzako, Ndoutabe Modjirom, Olushayo Oluseun Olu, Sylvester Maleghemi

**Affiliations:** 1 World Health Organization Country Office, Juba, Republic of South Sudan; 2 Ministry of Health, Juba, Republic of South Sudan; 3 Present Address: World Health Organization Regional Office for Africa, Brazzaville, Congo

**Keywords:** Circulating Vaccine Derived Polio Virus type 2, cVDPV2 outbreak, Descriptive epidemiology, Outbreak response, Chronic humanitarian, And conflict settings, mOPV2, South Sudan

## Abstract

**Background:**

In this study, we describe the epidemiological profile of an outbreak of the circulating Vaccine Derived Polio Virus type 2 in South Sudan from 2020 to 2021.

**Method:**

We conducted a retrospective descriptive epidemiological study using data from the national polio/AFP surveillance database, the outbreak investigation reports, and the vaccination coverage survey databases stored at the national level.

**Results:**

Between September 2020 and April 2021, 59 cases of the circulating virus were confirmed in the country, with 50 cases in 2020 and 9 cases in 2021. More cases were males (56%) under five (93%). The median age of the cases was 23.4 ± 11.9 months, ranging from 1 to 84 months. All states, with 28 out of the 80 counties, reported at least one case. Most of the cases (44, 75%) were reported from five states, namely Warrap (31%), Western Bahr el Ghazal (12%), Unity (12%), Central Equatoria (10%), and Jonglei (10%). Four counties accounted for 45.8% of the cases; these are Gogrial West with 12 (20%), Jur River with 5 (8.5%), Tonj North with 5 (8.5%), and Juba with 5 (8.5%) cases.

The immunization history of the confirmed cases indicated that 14 (24%) of the affected children had never received any doses of oral polio or injectable vaccines either from routine or during supplemental immunization before the onset of paralysis, 17 (28.8%) had received 1 to 2 doses, while 28 (47.5%) had received 3 or more doses (Fig. 4). Two immunization campaigns and a mop-up were conducted with monovalent Oral Polio Vaccine type 2 in response to the outbreak, with administrative coverage of 91.1%, 99.1%, and 97% for the first, second, and mop-up rounds, respectively.

**Conclusion:**

The emergence of the circulating vaccine-derived poliovirus outbreak in South Sudan was due to low population immunity, highlighting the need to improve the country’s routine and polio immunization campaign coverage.

## Introduction

The trivalent Oral Polio Vaccines (tOPV) are attenuated vaccines used globally to prevent and eradicate the poliovirus. They were used for over three decades until their synchronized withdrawal globally in 2016 [1, 2]. The Global Polio Eradication Initiative (GPEI) recommended them as the vaccine of choice for polio eradication, particularly in developing countries, because of their advantage in developing mucosal immunity, affordability, and ease of administration [3, 4]. As a result of their widespread use, the number of wild polioviruses (WPV 1, 2, and 3) cases have declined by over 99.9% from the 350,000 cases reported at the eradication initiative’s launch in 1988 [5, 6]. Currently, WPV1 is endemic in only two countries, Afghanistan and Pakistan. However, a progressive reduction in WPV1 cases has been noted in the last two years [7, 8]. In 2022, only 2 and 20 cases of WPV1 were reported from Afghanistan and Pakistan, respectively [9]. WPV 2 and 3 have been eradicated globally since 2015 and 2019 respectively, averting over 16 million infections of these viruses [10]. The WHO African Region (AFRO), which once contributed the highest poliovirus caseload globally, was officially declared WPV-free in August 2020 [11–13]. Nonetheless, the recent importation of WPV1 from Pakistan into the AFRO Region has added pressure to the strained system due to the ongoing Vaccine Derived Polio Virus outbreaks [14]. In 2020, a total of 21 countries were affected by the circulating Vaccine Derived Polio Virus type 2 (cVDPV2) outbreak, while from January 2020 to June 2021, there were active transmissions of cVDPV2 in 28 countries of the African region [15, 16].

Vaccine Derived Polio Virus (VPDV) outbreaks can emerge in areas with low population immunity [17]. There are three classes of VDPVs, namely Immune-Deficiency Poliovirus type 2 (iVDPV2) when it is associated with immunodeficiency, ambiguous poliovirus type 2 (aVDPV2) when found in an individual not immunocompromised or circulating, and circulating type poliovirus (cVDPV) when there is person to person community transmission [18, 19]. Of the three VDPV viruses, cVDPV2 is the most important as it causes frequent outbreaks, while iVDPV rarely persists [20]. Although other outbreaks do occur (cVDPV1 and 3), cVDPV2 outbreaks are the most reported in communities with low population immunity [21–23], with several outbreaks of cVDPV2 reported mainly in the African Region since the synchronized withdrawal of the tOPV globally in 2016 [24]. This is largely due to the reduction in the population immunity as a result of type 2 withdrawal from the routine immunization service [15, 16, 25–27]. Globally, 27 and 23 countries were affected by the cVDPV2 outbreak in 2020 and 2021 respectively, with 77% of the countries based in the AFRO Region [18, 27, 28]. The low coverage of the Injectable Polio Vaccine (IPV) in many countries has also contributed to these outbreaks [21, 29].

Achieving the polio eradication goal has become more challenging at this final stage of the programme as poliovirus is now located in hard-to-reach, inaccessible, and conflict areas and where vaccine hesitancy is an issue leading to delayed detection and response. To this end, GPEI developed an endgame strategy from 2022 to 2026 with two major goals. First to permanently interrupt all WPV transmission in endemic countries and second to stop all cVDPV2 transmission and prevent outbreaks in non-endemic countries by 2023 with the eventual declaration of polio eradication and global certification in 2026 [30]. To achieve these goals, a global surveillance action plan was developed to scale up the surveillance system’s sensitivity to detect and interrupt poliovirus circulation. More importantly, the strategy emphasizes cutting down on logistics challenges and the turnaround time of laboratory results for quicker response [31].

South Sudan withdrew tOPV from the routine immunization system and introduced IPV1 in 2015 and the bi-valent vaccine (OPV1,3) in 2016 [32, 33]. The country has remained WPV-free since 2009 but was amongst the last four countries in the WHO African Region (AFRO) to be certified WPV-free, resulting in WPV-free certification of the AFRO Region in August 2020 [11, 13, 34].

In late 2020, the country was notified of cVDPV2 cases, which eventually spread nationwide and was declared as an outbreak. This study describes the outbreak and provides evidence-based information that could be used to improve the response to future outbreaks in the country and others with similar settings. The study also documents the challenges faced and lessons learned from the outbreak response.

## Methodology

### Study design

Using secondary data, we conducted a retrospective descriptive epidemiological study of the cVDPV2 outbreak in South Sudan from September 2020 to April 2021. The data was obtained from the national polio/AFP surveillance, the cVDPV2 outbreak investigation reports, and the vaccination coverage survey databases that were stored and available at the national level. We also reviewed the reports of the outbreak response to synthesize the timelines, issues, and challenges associated with the investigation and response.

### Study setting

South Sudan, the youngest country in the world, is situated in East-Central Africa. It is bordered by Ethiopia in the east, Kenya in the southeast, Uganda in the south, the Democratic Republic of the Congo in the southwest, the Central African Republic in the west, and Sudan in the north. These borders are porous, allowing for the movement of people to and from the neighboring countries for cultural, commercial, and security reasons. The country has an estimated 2020 population of 13.8 million, 88% of whom live in rural areas. The population is predominantly young, with 47% estimated to be under 15 years. Administratively, the country is divided into 10 states and 3 administrative areas which are further sub-divided into 80 counties.

The country has experienced a chronic humanitarian crisis due to civil wars, inter-ethnic conflicts, drought and food insecurity, recurrent flooding, and disease outbreaks. This has resulted in displacement and disruption of social services, including the public health system. As of 31 December 2020, 7.5 million people were reported to need humanitarian assistance, of which 3.4 million were internally displaced to neighboring countries [35]. These have negatively impacted the polio surveillance, immunization, and other health service delivery systems [36].

### Security status

The high level political armed conflict that brooked out in 2013 continued until the present time in a different form. The protracted armed conflict in South Sudan, characterized by a complex web of political, ethnic, and regional tensions, has resulted in devastating consequences on the healthcare system and displaced several communities. The escalation of this conflict into full-blown civil wars in 2013 and 2016 further decimated the nation’s fragile health system. These wars and the pervasive inter-ethnic clashes resulted in the looting and destruction of vital health infrastructure, including cold chain assets, which significantly hampered the delivery of life-saving vaccines, including those against polio with an accumulation of susceptible zero-dose children.

### Sources of data

#### The South Sudan Acute Flaccid paralysis (AFP) surveillance database

In line with the GPEI, South Sudan operates an AFP surveillance system. This system detects, notifies, investigates, and verifies AFP cases in children under 15 years of age or in any person that a clinician suspects of having poliomyelitis using various case definitions (Table 1) at the community and health facility levels. The surveillance system is monitored through two main AFP surveillance indicators among many others: the non-polio AFP (NP-AFP) rate and the stool adequacy rate (Table 1). A third indicator, the non-polio enterovirus rate (NPENT), is also used for measuring the reverse cold chain system (Table 1).

**Table 1:** Case definitions

**Definitions**	
**AFP case:** An AFP case is a child < 15 years of age presenting with sudden onset of flaccid paralysis or muscle weakness due to any cause or any person of any age with paralytic illness if clinicians suspect poliomyelitis.	
**Confirmed polio case:** A suspected AFP case with poliovirus isolation from a stool sample.	
**Compatible **is an AFP case with inadequate samples and residual paralysis at 60 days follow-up investigation, and reviewed and classified by the National Polio Expert Committee (NPEC)	
**Non-polio AFP cases:** A suspected AFP case with no poliovirus isolation from a stool sample or inadequate case reviewed and discarded by the NPEC.	
**Non-polio AFP rate: **“number of discarded as NP-AFP cases in children < 15 years, divided by population < 15 years old multiplied by 100,000 per year”.	
**Stool adequacy:** “the number of AFP cases with two stools collected ≤ 14 days after paralysis of onset, collected ≥ 24–48 hours apart, and arriving in “good condition at the laboratory divided by a total number of AFP cases, and multiplied by 100” Good condition” means that upon arrival: There is a temperature indicator (showing < 8 °C) in the container, with no desiccation or leakage and proper documentation [37]	
**Non- poliovirus Enterovirus:** the other viruses present in stool apart from the poliovirus. Its presence in stool is an indication of adequacy of the stool sample	
**Discarded cases **are non-polio AFP cases classified by the National Expert Committee after an in-depth review of the cases that exclude all WPV, VDPV, and compatible cases.	
**Inadequate cases:** Cases detected over 14 days from the date onset of paralysis and arrival of the stool specimens to the laboratory in bad condition.	
**Investigation of AFP cases:** An initial investigation of suspected AFP cases conducted by health workers and verified by trained field staff, using a standard case investigation form to capture demographic, clinical, and epidemiological information with 60-days follow-up investigation for inadequate cases.	
**Final classification**: All reported inadequate AFP cases classified by the National Polio Expert Committee (NPEC) supported by the Secretariat. All adequate cases are classified automatically using a virological classification scheme with final approval of expert committee.	

Identification of persons who meet the case definition of AFP triggers the collection of two stool samples 24 h apart within 60 days of the onset of the illness. The stool samples are transported by air from the field location to the National Public Health Laboratory (NPHL) in the capital, Juba using a reverse cold chain method. A case-based form that includes clinical and epidemiological information is completed for every AFP case. Data from these forms are entered into the national polio/AFP database and kept at the NPHL with a copy kept at the WHO country office in Juba. The stool samples are then transported by air to the Uganda Virus Research Institute (UVRI), a WHO-accredited polio laboratory where laboratory analysis is conducted.

### Epidemiological investigation of cVDPV2 outbreak

Specific outbreak investigations were conducted for every confirmed cVDPV2 case by different teams. The data for this retrospective analysis includes all confirmed cVDPV2 cases, their contacts (siblings or other children living in the household), and children less than 5 years old living in the village where the confirmed cases were detected and investigated. The investigation was conducted using a standardized WHO investigation form [38]. During this outbreak, a mix of state and national investigators from different agencies with varying expertise were deployed to investigate the index case, while state teams investigated the subsequent cases. In some instances where there was insecurity, the local investigation team carried out the investigation using the standard investigation form with remote support from state and national teams. The case investigation includes two components. The first part of the investigation evaluates the case clinically, which includes eliciting clinical signs and symptoms to better relate the case to laboratory findings. The second part evaluates the epidemiological information to identify the risk factors for the spread of the outbreak such as travel history, vaccination status, contact history with others, etc.

As part of this investigation, a detailed assessment of the surveillance and routine immunization performance indicators was conducted including a review of the outcome of previous supplemental immunization activities. The aim was to establish the sensitivity of the surveillance system and routine immunization performance, including the cold chain status in all nearby health facilities. Furthermore, a retrospective review of records for unreported cases in health facilities and nearby communities was conducted.

### Laboratory analysis

In the laboratory, the stool sample is cultured to detect poliovirus, with a sequential analysis of the sample to come up with the result, and these include intratypic differentiation, and genome sequencing if the sample is positive. The method employed for the detection of poliovirus is the reverse transcription-polymerase chain reaction (RT-PCR). This technique is utilized to identify and differentiate the three types of polioviruses (type 1, type 2, and type 3) based on their genetic material. RT-PCR is conducted to amplify specific regions of the poliovirus genome present in the collected samples. The presence of the virus is confirmed by analyzing the fluorescence signals generated during the amplification cycles. These signals allowed for the determination of cycle threshold (Ct) values, indicating the initial viral RNA concentration in the samples [39]. UVRI notifies the Ministry of Health once the laboratory results are ready, and the Ministry enters the results into the national polio/AFP database to ensure data completeness.

For every reported AFP case, senior WHO and MOH EPI officers routinely conduct detailed case investigations. This investigation includes the follow-up of inadequate cases, which are cases that were reported and investigated after 14 days of the onset of paralysis or for which the stool samples are inadequate. The National Polio Expert Committee (NPEC), which meets every quarter classifies all inadequate cases and endorses adequate cases that the Secretariat has classified. All reported cases of AFP are entered into the national database and lab results are updated as they become available. Weekly data harmonization is conducted to avoid discrepancies among different levels.

### Rapid assessment of the vaccination coverage survey

Immunization is one of the critical areas that was included in the investigation, and we retrospectively reviewed the secondary data. In this respect, a rapid assessment of the immunization profile of children in areas where cVDPV2 cases were detected was assessed. In this regard, 30 households with at least one child less than 5 years of age were assessed. Information on the child’s age in months and the number of doses of OPV and IPV immunization received was collected. The immunization status was determined by review of vaccination cards, history, and facility records as applicable.

### Data collection and analyses

For this study, we retrospectively analyzed the secondary data stored at the national level. We extracted clinical and epidemiological data based on WHO’s definition of cVDPV2 from the national polio/AFP databases and we reviewed outbreak investigation report that was also stored and available at the national level. These were entered into a Microsoft Excel sheet. The inclusion criteria are all children under 15 years whose polio laboratory results tested positive for cVDPV2, and the exclusion criteria are all children under 15 years whose results were negative. Data on the performance of the polio surveillance system was extracted and calculated from the national polio/AFP surveillance database. The immunization status of healthy children that was assessed by the different investigating teams was combined and entered in to Microsoft Excel and exported to EPI info version 7.2.5 for analysis.

We conducted a retrospective descriptive epidemiological analysis of the clinical and epidemiological data using frequencies, tables, and graphs. We calculated the non-polio AFP rate (NP-AFP) and stool adequacy using the global standard formula [40, 41].

## Results

The index case and the origin of the outbreak.

The country received a notification from UVRI about isolating Polio Virus Type 2 (PV2) from three AFP samples on 4 September 2020. Two of these cases occurred in Jur River county in Western Bahr el Ghazal state, while the third case was reported from Tonj North County in Warrap state. The outbreak was confirmed on 17 September 2020, 96 days after the second sample was collected and 23 days after the sample arrived at the reference lab.

The index case was a male child aged 23 months from Tharkueng village in Jur River county of Western Bahr el Ghazal (WBG) with a date of onset of paralysis on 10 June 2020. The child had received three doses of bOPV and a dose of IPV from a combination of routine immunizations and campaigns. His two contacts were also positive for cVDPV2. The index case indicated that he had traveled to neighboring Warrap state before the onset of paralysis and stayed there for one month, but his parents claimed that they had no contact with anyone who had recently traveled outside of the country. One week after his return, he developed a sudden onset of asymmetric paralysis of the right lower leg, and he was taken for medical consultation at a private hospital in Wau, with the AFP surveillance focal person of the state notified the same day. The surveillance focal person investigated the case the same day and collected two stool samples at 24-hour intervals on 13 and 14 June 2020. The samples arrived at UVRI on 25 August 2020.

The sequencing analysis result of the index case from the South African National Communicable Disease Institute (NICD) indicated a cVDPV2 outbreak with 19 nucleotides divergence from Sabin 2, closely related to the virus circulating in Chad. Subsequent genetic analyses of confirmed cases indicated a mix of importations that were closely related to the virus circulating in Ethiopia (2%), Sudan (13%), and Chad (16%), with local transmission accounting for 69% of the total cases.

### Epidemiological profile and evolution of the outbreak

Following the confirmation of the cVDPV2 outbreak on 17th September 2020, the Ministry of Health South Sudan declared the outbreak as a “Public Health Emergency’’ on 18 September 2023 and sent an alert letter to all states informing them about the outbreak and requesting the need for enhancing surveillance. On the same date an Incident Management System (IMS) was established with the Director for Preventive Medicine assigned as a chair. Terms of Reference and composition of IMS for the outbreak control were identified and the first response meeting convened on 18th September 2020.

**Fig. 1:**
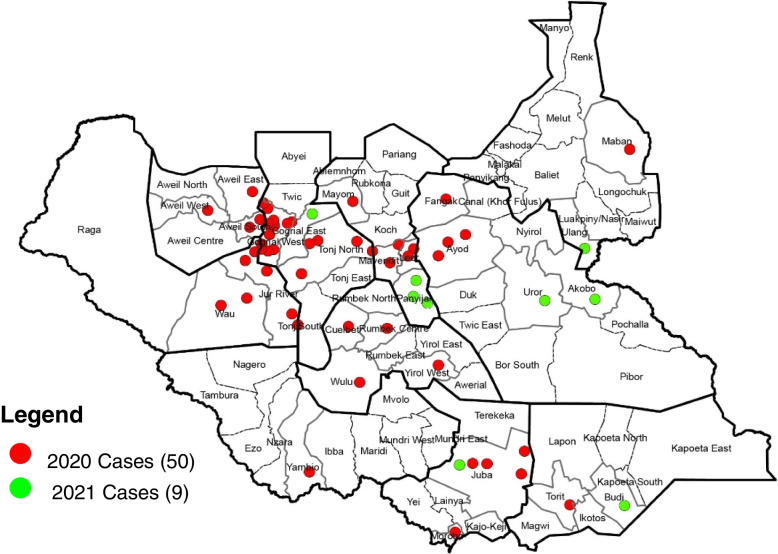
Geographical distribution of cVDPV2 cases in South Sudan: 2020-2021

Between September 2020 and April 2021, 59 cases of cVDPV2 were confirmed with 50 cases in 2020 and 9 cases in 2021. Most cases were male (56%) and under five years of age (93%). The median age of the cases was 23.4 ± 11.9 months, ranging from 1 to 84 months. Regarding geographical distribution, all ten states and 28 out of 80 counties reported at least one case. Most of the cases (44, 75%) were reported from five states, namely Warrap (31%), Western Bahr el Ghazal (12%), Unity (12%), Central Equatoria (10%), and Jonglei (10%). Four counties accounted for 45.8% of the cases; these are Gogrial West with 12 (20%), Jur River with 5 (8.5%), Tonj North with 5 (8.5%), and Juba with 5 (8.5%) cases (Fig. 1).

**Fig. 2:**
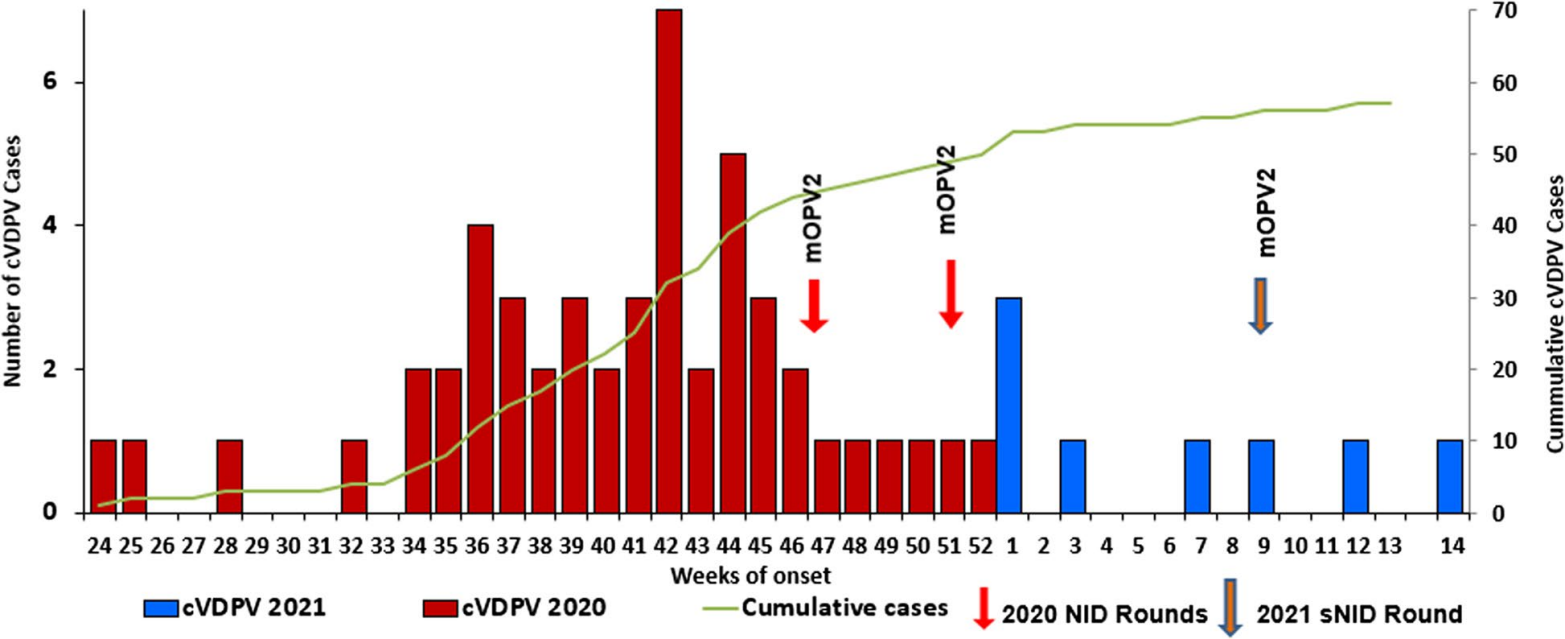
The epidemiological curve of cVDPV2 outbreak in South Sudan: 2020-2021

The index case was diagnosed during epidemiological week 24, 2020 and subsequently, only 4 cases were detected from Epi weeks 24 to 32 of 2020. However, from Epi weeks 34 to 46, the outbreak peaked, and 42 cases (71%) were detected. One case per week was reported from then until the end of 2020, with sporadic cases reported in 2021. The outbreak trend indicates rapid transmission with 98% of the cases reported within 28 weeks of the index case date onset. The onset of the last case occurred in Panyijar county of Unity state, on 8 April 2021 (Fig. 2).

The epidemiological investigation that was conducted indicated that none of the confirmed cases had a history of contact with someone outside the country or a recent travel history to outbreak-affected areas outside the country. The clinical investigation of cases showed that 95% and 47% had a fever and asymmetric paralysis, respectively. The laboratory results of 13 out of 48 household contact samples were positive for cVDPV2, with the nucleotide change ranging from 17 to 24 nucleotides.

Regarding surveillance, all States met the two key AFP surveillance indicators, with the NP-AFP rate above 3.2/100,000 children < 15 years old and stool adequacy of 80% and above for 2019, 2020, and 2021 except for Jonglei State that had less than 80% stool adequacy in 2019 and 2020 and Upper Nile State in 2020. On the other hand, the analysis of surveillance performance indicators at the county level indicated that 19 and 2 counties in 2020 and 2021 respectively were below the required target of 3/100,000 children < 15 years, while 22 and 14 counties were also below the target for stool adequacy in the same period respectively (Table 2).

**Table 2:** Polio surveillance performance indicators by state pre and during the outbreak in South Sudan

S/N	State	No of Counties	Counties affected	Total cVDPV2 cases reported		Source of cVDPV2		Non-polio AFP rate Per 100,000 population < 15 yrs.)			State Stool Adequacy Rate			No. of compatible cases 2019–2020	Percentage of counties with OPV3 coverage > 80% in 2019 (preoutbreak)	No. of silent counties in 2019	Both indicators meeting 2019–2021	Counties meet > = 3/100,000 children < 15 years in 2020	Counties meet > = 3/100,000 children < 15 years in 2021
				2020	2021	AFP samples	ES samples	2019	2020	2021	2019	2020	2021						
1	Central Equatoria	6	2	5	2	7	5	3.6	3.8	6.3	93	97	95	0	17	1	10	3	6
2	Eastern Equatoria	8	2	1	1	2	0	4.3	6.8	6.5	93	83	94	1	0	0	8	6	8
3	Jonglei	11	4	4	2	6	0	4.1	4.2	8.6	72	54	83	10	0	1	7	8	11
4	Lakes	8	4	4	0	4	0	9.8	7.5	5.6	94	95	94	0	38	0	6	8	8
5	Northern Bahr El Ghazal	5	3	4	0	4	0	5.8	4.4	5.8	82	81	89	2	20	0	6	2	5
6	Unity	9	4	5	2	7	0	11.2	9.1	14.3	85	80	93	4	0	0	6	7	9
7	Upper Nile	9	2	1	2	3	0	5.5	6.3	8.5	88	78	97	2	8	1	3	10	11
8	Warrap	7	3	17	1	18	0	6.7	8.3	9.0	96	91	92	2	14	0	3	5	7
9	Western Bahr el Ghazal	3	3	7	0	7	0	11.6	12.4	16.1	93	94	85	0	0	0	3	3	3
10	Western Equatoria	10	1	1	0	1	0	8.2	7.1	7.9	100	91	92	1	70	0	3	8	9
	National	80	28	49	10	58	5	6.3	5.6	8.2	90	84	91	22	18	3	55	60	77

**Fig. 3:**
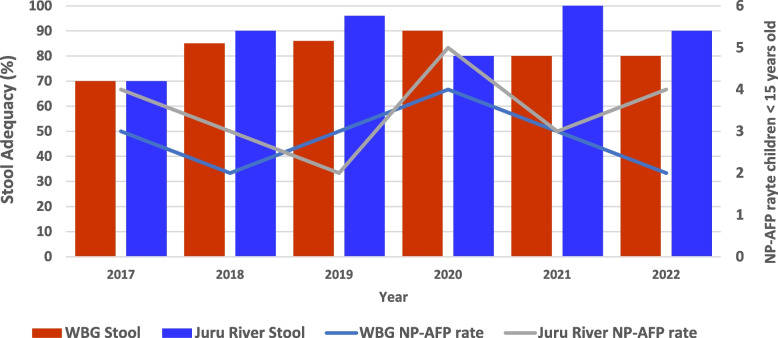
The performance of AFP surveillance indicators in Western Bahr el Ghazal State and Jur River County of South Sudan: 2017-2022

The NP-AFP rates and stool adequacy of Western Bahr el Ghazal State and Jur River County, where the index case was reported were above the standard surveillance performance indicators before and during the outbreak (Fig. 3).

**Fig. 4:**
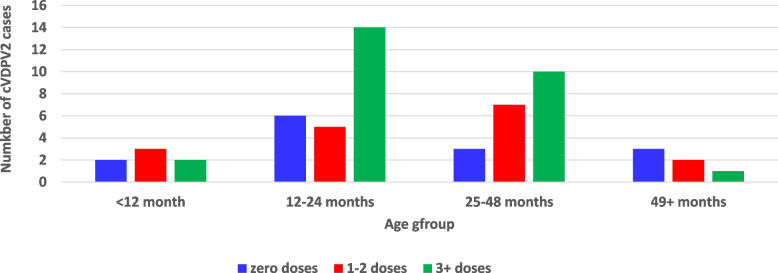
The OPV vaccination status of cVDPV2 cases by age group in South Sudan: 2020-2021

The immunization history of the confirmed cases indicated that 14 (23.7%) of the affected children had never had any doses of OPV or IPV either from routine or during supplemental immunization before the onset of paralysis, 17 (28.8`%) had received 1 to 2 doses, while 28 (47.5%) had more than 3 doses (Fig. 4).

**Fig. 5:**
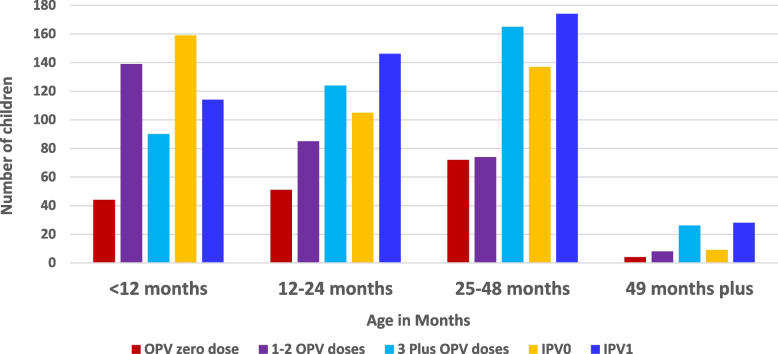
The OPV vaccination status of healthy children under five years at the community level in South Sudan: 2020 - 2021

At the community level, 882 healthy children under five years were assessed for their vaccination profile, of which 171 (19.4%) had never had any dose of OPV (zero doses), while 306 (34.7%) had gotten 1 to2 doses and 405 (45.9%) had received more than 3 doses. For IPV, 410 (47%) of the healthy children had received one dose, and the rest (53%) had not received any dose (Fig. 5).

### Outbreak response

**Fig. 6:**
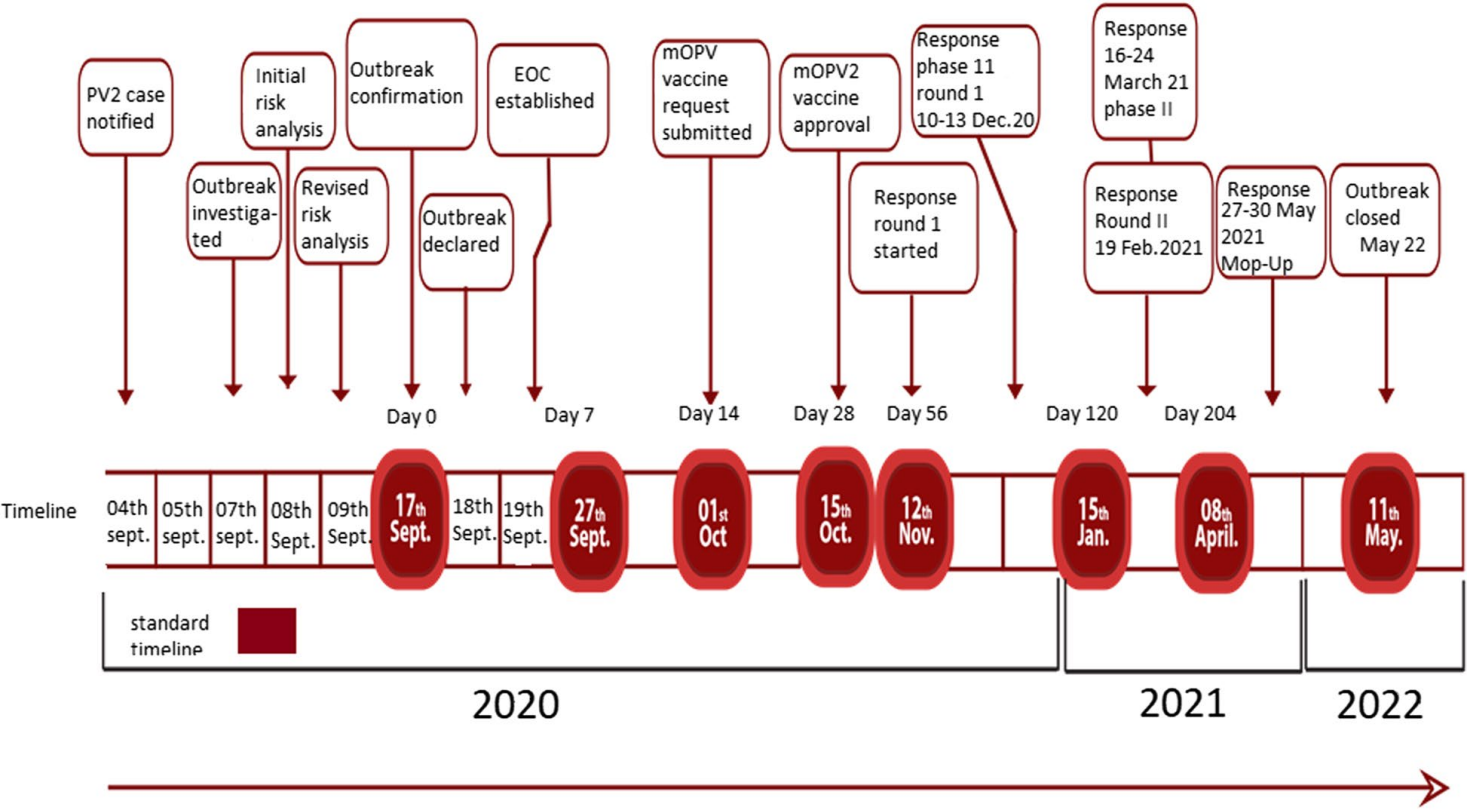
The Timeline of cVDPV2 outbreak response activities in South Sudan: 2020-2021

The timeline of various response activities that were conducted is presented in Fig. 6. A preliminary risk assessment was developed and submitted to the Outbreak Preparedness and Response Group (OPRG) on 8 September 2020, while the final risk assessment was completed on 18 September 2020, after incorporating the initial risk assessment comments made by OPRG. The country team based on the initial risk assessment recommended all 80 counties for mOPV2 response campaigns, but the OPRG insisted to focus on 45 counties where the case was identified and surrounding counties. However, before the response campaign, an additional 12 cVDPV2 cases from four more states were identified and the risk assessment was updated and resubmitted on 18 October 2020, with the whole country (80 counties) targeted for the response using mOPV2 vaccine.

Two nationwide immunization response campaigns were undertaken in a phased manner with a mop-up campaign done in counties that performed poorly and failed to pass the Lot Quality Assessment (LQA). The first round of the vaccination campaign was implemented in four phases and started on 10 November 2020. Seventy-five (75) counties implemented at least 1 round by 11 December 2020, while 4 counties implemented the campaign by March 2021. One county (Tonj East) was covered during the mop-up round with no second round conducted. The second round of the campaign was conducted in three phases, which started 8–11 December 2020 (1st phase), 16–19 February 2021 (2nd phase), and 15–18 June 2021 (3rd phase) and reached 6, 68, and 4 counties, respectively. A mop-up campaign was conducted from 25 to 27 May 2021, covering 18 counties in nine states. The immunization campaigns of the first and second rounds targeted 2,708,729 and 2,610,684 under-five children, respectively, with the average administrative coverage data for the first and second rounds being 91.1% and 99.1%, respectively (Table 3).

**Table 3:** Coverage of mOPV2 vaccination campaigns in response to the cVDPV2 outbreak in South Sudan: 2020–2021

State	Round 1		Post Campaign Evaluation Coverage Round 1		Round 2		Post Campaign Evaluation Coverage Round 2		Campaigns		Mop up
	Administrative coverage	IM coverage ( In house)	In House(%)	Outside House(%)	Administrative coverage	IM coverage ( In house)	In House(%)	Outside House (%)	1st Round	2nd round	
Central Equatoria	85	93	93	90	93	88	88	81	Phase I from 10–13 Nov 20 20 (44counties) Phase II: 8–11 Dec. 2020 (31 counties) (Phase III) 16–22 Mar 2021 (4 counties) Phase IV 27–30 May 2021(1 county)	Phase I 8–11 Dec 20 20 (6 counties) Phase II: 16–19 Feb 2021 (68 counties) Phase II 15–18 June 2021 (4 counties)	27–30 May 2021 in 18 counties 97% coverage
Eastern Equatoria	89	94	94	88	90	89	89	83			
Jonglei	84	NA	NA	NA	90	77	77	84			
Lakes	100	90	90	88	106	91	91	88			
Northern Bahr El Ghazal	111	92	92	94	120	93	93	96			
Unity	79	87	87	93	96	87	87	89			
Upper Nile	90	98	98	90	89	87	87	91			
Warrap	99	94	94	86	116	89	89	90			
Western Bahr El Ghazal	86	76	76	89	90	88	88	98			
Western Equatoria	92	87	87	88	95	97	97	91			
National	91.1	90.9	90.9	89	99.1	88.2	88.2	87.7			

The independent in-house monitoring results for the first and second rounds showed coverage rates of 90.1% and 88.2%, respectively, while mop-up targeted 847,400 under-five children with administrative coverage of 97%. LQA was conducted in all rounds, including mop-up, and based on the results, 12 out of 46, 13 out of 37 counties, and 12 out of 15 counties were included in the first, second, and mop-up rounds, respectively. The administrative coverage for the campaign at the sub-national level for both rounds was below 95%, except for three and four states that achieved 95% and above in the first and second rounds, respectively, while the Post Campaign Evaluation (PCE) indicated that 8 out of the 9 states where PCE was conducted were below 95% coverage in the first round, while in the second round, 8 of the 10 states were below 95% coverage.

As a result of the conflict, response activities have been hindered. First and foremost, it is important to note that the response was phased instead of a nationwide campaign and took a longer time to complete the first round. In this regard, the first response round was completed after 4 months since confirmation of the outbreak, and this may have contributed to transmission and further spread. Tonj East County campaign's was delayed due to intense active tribal fighting in the county and was covered only with a round during a mop-up round after the situation had calmed down months after. Four counties in the Sobat corridor had the response campaign in March 2020 instead of an immediate response in Dec 2020. In addition, the coverage quality of the LQA survey has indicated quality issues, and only 12 out of 46 (26%) and 13 out of 37 (35%) counties passed in the first and second round LQA assessment respectively however the mop-up round 12 out of 15 (80%) of the counties passed the LQA assessment.

## Discussion

This study describes the first outbreak of cVDPV2 after the tOPV switch in South Sudan and the response to it. The findings showed that the outbreak was widespread and fast spreading, affecting 28 counties across all ten States of the country. Due to active fighting, Tonj East was covered with only one round of response during mop-up many months after other counties had done the planned 2 rounds. The highest number of cases were reported from Gogrial West, Jur River, Tonj North and Juba Counties, while most of the cases were reported within 17 weeks of the onset of the outbreak. Furthermore, nearly a quarter of the affected children had never received any doses of OPV or IPV, while slightly more than a quarter of the children had received 1–2 doses, and nearly half of the confirmed cases received 3 plus doses highlighting the role of high immunization coverage in stopping such outbreaks. The low immunization coverage stemmed from poor access to health facilities with many of the facilities not functional due to destruction attributed to the prolonged conflict. Most of the confirmed cases were children under 5 years old. As part of the response, vaccination campaigns were conducted in two rounds (phased approach), and a mop-up was conducted in poor performing and insecure and high-risk areas of the country.

The first response campaign started in a phased manner albeit these response activities were delayed due to the protracted conflict, natural disasters, and the COVID-19 pandemic in the country. In response to these challenges, the MOH collaborated with the implementing partners present in the conflict affected areas to take responsibility to vaccinate eligible children.

Insecurity and active tribal conflict played a significant role in delaying the implementation of the response campaign, and it took considerable time to complete the first round in the targeted areas. This resulted in a lack of trust between the government and the local population, which further hindered the implementation of the campaign. The insecurity and active tribal conflict led to an increase in civilian casualties, which resulted in the local population’s reluctance to cooperate. Following the confirmation of the outbreak, the first round, which targeted 75 counties was completed in December 2020, and the global standard of 28 days was not met [42]. This delay in response caused the outbreak to spread further and the conflict with the resultant destruction of infrastructure made it difficult for health workers to access the affected areas and provide the necessary response. In addition, the first response national campaign could not be implemented in one go throughout the country and was phased out over time. The implementation of the campaign was purposely delayed in five counties until the security situation had calmed down. For example, the Tonj East County underwent active tribal conflict during the planned period, and this led to a postponement to some other time until the situation was calm, and this led to the county being covered with only one round of response. On the other hand, because of some form of conflict or the consequence of the previous political conflict, the four counties in the Sobat corridor were not able to go with the national plan, with the MoH mandating the local health implementing partners who are knowledgeable on the local security situation of the area to conduct the campaigns. This contributed to the delay in implementing the response campaign as per the set standard. The conflict has also contributed to the poor surveillance sensitivity which resulted in a lack of access to health services, as well as an increase in health risks for vulnerable populations. Additionally, the conflict impaired our ability to conduct active case searches and sensitization, including monitoring and tracking the quality of the campaign. In 2020 and 2021, the inter-communal conflict in South Sudan affected campaign activities, including surveillance achievement although progress was noted in the second year of the outbreak. This is mostly related to limited access. Although many positive things have happened since the revitalized peace agreement, none government intercommunal fighting and conflict increased in scale and intensity for the years 2021 and 2021 [42].

The floods in 2020/21 made it difficult for health workers to reach the displaced populations as they moved uphill which required long hours of volunteers to travel on foot and required hiring of boats to address these challenges. During the two consecutive years, nearly one million population were displaced due to flooding [43, 44]. This left most areas of the country inaccessible as most public structures and health services were disrupted compounding the challenges of responding to the polio outbreak. Tonj East county despite the ongoing security issue conducted only a round of campaign due to discussions and interventions with the political leadership and security agents [45].

The wide and fast-spreading nature of the outbreak may be attributed to the low population immunity [46–48] and widespread population movements within the country. The phased approach, to the response may also have contributed to the spread of the virus. This approach was necessary for two reasons. Firstly, the security situation wasn’t conducive to a nationwide campaign. Secondly, wider use of the mOPV2 vaccine would have created challenges in managing the vaccine and retrieving the leftover vaccine vials after the campaign, which is the standard for mOPV2 use [49]. As a result, the phased approach, was used for practical reasons.

The outbreak was not unexpected; the switch from tOPV to bOPV in 2016 in the country allowed for a build-up of a cohort of children susceptible to the type 2 poliovirus for five years. This was further complicated by the low immunization coverage including IPV across the country, especially in the traditionally conflict-affected states of Unity, Jonglei, and Upper Nile. The routine immunization coverage in the country has been reported consistently low according to WHO and UNICEF estimates, with the country being consistently below 50% for many years [22]. Likewise, the administrative immunization coverage (OPV3) also reported a decline from 77% in 2010 to 56% in 2020 [50], which is even lower in the conflict-affected states that reports below 50% [42, 43]. The immunization coverage survey conducted in 2017 also indicated low IPV coverage of 20% in the traditional conflict-affected states [50, 51]. The reduction of population immunity may also be due to the reduction of the number of supplemental immunizations which were once conducted four times a year, but were reduced in frequency in 2018 and 2019 with only one round done in 2020. This fact is corroborated by the study findings which showed high zero dose children (22%) for OPV and IPV among the children assessed in the surrounding communities, with more vaccinated among the confirmed cases. This finding is inconsistent with similar studies conducted in Syria, DRC, Yemen, and Nigeria including the findings in China where cVDPV1 outbreaks in 2020 were more zero dose children among confirmed cases [52–58].

The country team had initially requested and planned for a nationwide response campaign, however, the OPRG advised a more limited intervention encompassing 45 counties. While discussions ensured between the MoH and the OPRG, with delay in the response, the outbreak persisted spreading to other counties, eventually leading to the acceptance by the OPRG of the need for 2 rounds of nationwide polio campaigns. This experience underscores the need to recognize the country team as best placed to determine the scope of outbreak responses. Furthermore, the deliberations surrounding the type of vaccine to use (trivalent or monovalent polio vaccine) highlighted the importance of strategic vaccine choices. While seeking to make the most suitable decision for the context, these discussions inadvertently contributed to additional response delays. Given this backdrop, our recommendation to empower the country team to make agile decisions that align with emerging realities aimed at enhancing the responsiveness and effectiveness of the response efforts cannot be overemphasized.

The study findings indicating that more than three-quarters of cases were under the age of 5 years is consistent with the findings of many other studies [59, 60]. Although the county and the state where the index case was detected met the two main surveillance performance indicators before, during, and post-outbreak period; regular active case search visits remained inadequate in the state and county mainly due to logistic challenges and insecurity.

The high number of cases from Gogrial West County may be due to the Auto Visual AFP Detection and Reporting (AVADAR) project which was operating in the county during the outbreak period. AVADAR is a community-based surveillance system with a mandatory weekly report on a mobile platform. It also enables community informants to sensitize the community as videos are played weekly to remind the focal persons and the community to report on AFP cases. A study conducted in 2019 to assess the impact of AVADAR on AFP surveillance indicated that the AVADAR community-based surveillance system had improved the detection rate of AFP cases compared to non-AVADAR-implementing counties [61].

The population immunity against the type 2 vaccine is low due to the poor IPV coverage in those counties that reported a high number of cases which was the case in many other countries that reported such outbreaks [1, 52, 62].

The delayed outbreak detection (and response) was also due to the COVID-19 lockdown, which delayed the transportation of the stool samples from Juba to UVRI, Entebbe, and subsequently delayed the laboratory processes and the turnaround time of the results. South Sudan usually sends the samples within 3 days after arrival in Juba through air transport to UVRL however, with the shutdown of the country’s air space, the country resorted to road transport which could only occur when the land borders between the country and Uganda were opened, and the required documentations were obtained. This accounted for the time period of 96 days from when the second sample was collected, and the positive results were received which is much higher than the average of 59 days that was reported by a study that characterized 74 outbreaks of cVDPV2 from different countries. This highlights the significant role of surveillance sensitivity in timely detection and response [63].

The declaration of the outbreak by the South Sudan Ministry of Health was prompt and consistent with the global standard operating procedure released in May 2022, and compared with other outbreaks [64, 65]. Nevertheless, the outbreak response was late resulting in a longer period to interrupt the outbreak compared to the global standard of 120 days [66]. However, the interruption of the outbreak was achieved in a relatively shorter time compared to the outbreaks recorded in the Philippines, Cameroon, and Yemen [60, 67, 68]. In our study, the delay in interruption of the outbreak could be attributed to the non-implementation of the rapid response campaign within 14 days of confirmation of the outbreak, and the phased approach of immunization response during both rounds. The first round started on 10 November 2020 which was 54 days after the confirmation of the outbreak and was completed after six months on 30 May 2021 due to many reasons such as insecurity, flooding, and difficult terrain which limited access, the COVID-19 pandemic response, and other operational delays. The second round of the campaign was also delayed and completed in a phased manner as the first round, and most of the counties (68 counties) were covered six months after confirmation. Furthermore, the one-month standard implementation interval between the first and second rounds was not adhered to in all phases since the mOPV2 vaccine request, release, and arrival in the country was delayed due to logistics reasons.

Notably, the establishment and utilization of the Polio Emergency Operation Control played a pivotal role in guiding decisions. This centralized command structure, led by the Director General Directorate in charge of Primary Health Care in the MOH, serving as the incident manager, exemplifies the importance of using a cohesive framework during crises.

The experience also underscores the paramount significance of adaptability, community engagement, and where applicable MOH outsourcing of response campaign activities in hard-to-reach and insecure areas to local implementing partners who have established presence and systems in such areas.

Additionally, the Polio outbreak response highlighted the critical role of collaboration and coordination among stakeholders. This model of engagement, effectively employed through the Emergency Operation Control, facilitated alliances with international organizations and local partners which enhanced the reach and impact of interventions.

Furthermore, the experience emphasizes the value of data-driven decision-making and real-time monitoring. These practices ensured that the interventions remain relevant and that strategies can be adjusted promptly based on emerging trends, as guided by the centralized control structure.

The above findings should be interpreted in the context of two key limitations. First, the data on the risk factors for transmission of cVDPV2 was incomplete therefore multivariate analyses to determine associations could not be conducted. Second, most of the investigation reports and immunity profiling of children in the community were self-reported data that could contain interviewee bias.

## Conclusion

South Sudan grappled with its first cVDPV2 outbreak following the tOPV3 switch spanning from 2020 to 2021, primarily attributed to the introduction of the virus from Chad. The outbreak’s exacerbation was driven by the critical issues of low population immunity, stemming from inadequate immunization coverage, the fluid dynamics of population movement within the country, delayed response strategies, and the concurrent challenges posed by the ongoing COVID-19 pandemic. The lessons gleaned from the investigation and response to this outbreak underscore the pressing need for enhanced timeliness in the delivery of laboratory results and the establishment of expedited approval and vaccine release mechanisms to facilitate swift responses to cVDPV2 incidents.

Our findings led to a series of key recommendations. Firstly, we advocate for comprehensive improvements in routine immunization coverage, with a particular emphasis on the administration of OPV/IPV, prioritizing under-immunized and zero-dose children. This should involve periodic intensification and supplementary immunization activities to guard against any resurgence of cVDPV2 in the nation until the ultimate global certification of polio eradication.

Secondly, we propose affording greater autonomy to country teams that possess a deeper understanding of the local context, allowing them to make informed decisions regarding the target population, geographical scope, and vaccine selection. This measure aims to reduce or eliminate unnecessary delays in response efforts.

Thirdly, we recommend conducting thorough research on similar outbreaks to comprehensively investigate the associated risk factors, enabling a more targeted and effective response.

Finally, considering the logistical and operational challenges stemming from South Sudan’s persistent conflict situation, we urge the development, testing, and implementation of cVDPV2 contingency plans. These proactive measures are essential in anticipating and mitigating some of the challenges that may arise in the event of future outbreaks. This multi-faceted approach, encompassing immunization, surveillance hard to reach areas, decision-making, research, and preparedness, is essential to safeguarding the health of South Sudan’s population and advancing the broader global goal of polio eradication.

## Data Availability

Most data elements for this study are presented in the tables and figures. However, the complete study data is available upon request from the corresponding author.

## Abbreviations

AFP: Acute Flaccid Paralysis
bOPV: Bivalent Oral Polio Vaccine
IPV: Injectable Polio Vaccine
cVDPV2: circulating Vaccine Derived Polio Virus Type 2
VDPV: Vaccine Derived Polio Vaccine
aVDPV: ambiguous Vaccine Derived Polio Virus
iVDPV: Immuno Deficiency Vaccine Derived Polio Virus
mOPV2: Monovalent Oral Polio Vaccine 2
OPV: Oral Polio Vaccine
OPRG: Outbreak Preparedness and Response Group
PV2: Polio Virus type 2
tOPV: Trivalent Oral Polio Vaccine
VDPV2: Vaccine derived Polio Virus Type 2
WBG: Western Bahr Ghazal
WPV1,2,3: Wild polio virus type 1,2,3
